# Concordance Between Survey and Electronic Health Record Data in the COVID-19 Citizen Science Study: Retrospective Cohort Analysis

**DOI:** 10.2196/58097

**Published:** 2025-07-28

**Authors:** Elizabeth Crull, Emily C O'Brien, Pavel Antiperovitch, Kirubel Asfaw, Alexis L Beatty, Djeneba Audrey Djibo, Alan F Kaul, John Kornak, Gregory M Marcus, Madelaine Faulkner Modrow, Jeffrey E Olgin, Jaime Orozco, Soo Park, Noah Peyser, Mark J Pletcher, Thomas W Carton

**Affiliations:** 1 Department of Health Services Research, Louisiana Public Health Institute, 400 Poydras Street, Suite 1250, New Orleans, LA, 70130, United States, 1 5044954903; 2 Duke Clinical Research Institute, School of Medicine, Duke University, Durham, NC, United States; 3 Department of Epidemiology and Biostatistics, University of California, San Francisco, San Francisco, CA, United States; 4 Division of Cardiology, University of California, San Francisco, San Francisco, CA, United States; 5 Safety, Surveillance, and Collaboration, CVS Health, Blue Bell, PA, United States; 6 Medical Outcomes Management, Inc., Sharon, MA, United States

**Keywords:** electronic health records, self-report, COVID-19, data accuracy, data validation, EHR, cohort, cohort analysis, real-world data, concordance, internet-based, portal, participant, report, reported

## Abstract

**Background:**

Real-world data reported by patients and extracted from electronic health records (EHRs) are increasingly leveraged for research, policy, and clinical decision-making. However, it is not always obvious the extent to which these 2 data sources agree with each other.

**Objective:**

This study aimed to evaluate the concordance of variables reported by participants enrolled in an electronic cohort study and data available in their EHRs.

**Methods:**

Survey data from COVID-19 Citizen Science, an electronic cohort study, were linked to EHR data from 7 health systems, comprising 34,908 participants. Concordance was evaluated for demographics, chronic conditions, and COVID-19 characteristics. Overall agreement, sensitivity, specificity, positive predictive value, negative predictive value, and κ statistics with 95% CIs were calculated.

**Results:**

Of 34,017 participants with complete information, 62.3% (21,176/34,017) reported being female, and 62.4% (21,217/34,017) were female according to EHR data. The median age was 57 (IQR 42‐68) years. Out of 34,017 participants, 81.6% (27,744/34,017) of participants reported being White, and 79.5% (27,054/34,017) were White according to EHR data. In addition, 9.2% (3,124/34,017) of participants reported being Hispanic, and 6.6% (2,249/34,017) were Hispanic according to EHR data. Statistically significant discordance between data sources was detected for all demographic characteristics (*P*<.05) except the female category (*P*=.57) and the American Indian and Alaska Native (*P*=.21) and “other” race categories (*P*=.33). Statistically significant discordance was detected for the 2 COVID-19 traits and all baseline medical conditions except diabetes (*P*=.17). The starkest absolute difference between data sources was for COVID-19 vaccination, which was 48.4% according to the EHR and 97.4% according to participant report. Overall agreement was high for all demographic characteristics, although chance-corrected agreement (κ) and sensitivity were lower for the “other” race category (κ=0.31, sensitivity =26.6%), Hispanic ethnicity (κ=0.82, sensitivity=74%), and current smoker status (κ=0.54, sensitivity=49.4%). Specificity and negative predictive value (NPV) were higher than corresponding specificity and positive predictive value (PPV) for all baseline medical conditions. Sleep apnea had the highest sensitivity of all medical conditions (83.5%), and anemia had the lowest (32.8%). Chance-corrected agreement (κ) was highly variable for baseline medical conditions, ranging from 0.26 for anemia to 0.71 for diabetes. Overall and chance-corrected agreement between data sources for COVID-19 traits such as infection (84.6%, κ=0.34) and vaccination (51.0%, κ=0.05) was relatively lower than all other evaluated traits. The sensitivity for COVID-19 infection was 32.2%, and the sensitivity for COVID-19 vaccination was 49.7%. Although PPV for COVID-19 vaccination was 99.9%, the NPV was 5%.

**Conclusions:**

Results suggest the need for improvements to point-of-care capture of patient demographic traits and COVID-19 infection and vaccination history, patient education about their medical conditions, and linkage to external data sources in EHR-only pragmatic research. Further, these results indicate that additional work is required to integrate and prioritize participant-reported data in pragmatic research.

## Introduction

The advent of electronic health record (EHR) systems and internet-based study portals have modernized and streamlined pragmatic clinical research [1,2], defined as research that can be conducted in real-world settings with minimal change to clinical operation [3,4]. The use of patient- and study participant–reported data alongside EHR data is increasingly common in research and clinical practice to complement and validate EHR data sources [5,6]. Therefore, there is potential value in linking and comparing patient experience and outcomes data gathered from mailed surveys [7-9] and patient-facing web-based portals [10] with data extracted from EHRs. This is especially true for clinical concepts that are notoriously difficult to qualify using medical coding alone, such as mood, gastrointestinal disorders, and chronic pain [11].

EHR and participant-reported data each have significant limitations. EHR data are fraught with administrative error, incomplete mapping to clinical ontologies, lack of legacy health record data, and inability to extract important clinical information from unstructured physician notes [12,13]. Participant-reported data are subject to bias from social desirability [10], fatigue [14], and limited understanding of medical issues. How these limitations affect the reliability of different kinds of health-related information is of interest for this substudy. There is a particular need for understanding the reliability of health information related to COVID-19, especially because a large portion of home testing and vaccination occurred outside traditional health systems.

The COVID-19 Citizen Science Study (CCS) is a longitudinal digital cohort study designed to generate knowledge about participant-reported outcomes related to the COVID-19 pandemic [15]. The study linked participant-reported data with their corresponding EHR data, thus presenting an opportunity to analyze the concordance between these data sources. The purpose of this study was to assess the concordance of COVID-19–related outcomes, demographic characteristics, smoker status, and 12 common medical conditions.

## Methods

### Overview

Our study evaluated concordance between two data sources: (1) participant-reported data from a web-based patient portal for the CCS study and (2) participants’ corresponding EHRs, conforming to a common data model. Data from the participant-reported data source were converted first to a CSV format and then imported into SAS 9.4 software (SAS Institute) as datasets. Data from the participants’ EHRs were loaded along an extract-transform-load pipeline from the source EHR to a relational database management system and finally into SAS 9.4 software as well to enable comparison with participant-reported data.

### Study Recruitment

Our concordance assessment used participant-reported and EHR data collected as part of the CCS study (ClinicalTrials.gov identifier NCT5548803), which has been described in detail previously [15]. Participants were recruited from 7 major health systems in Texas, Louisiana, Mississippi, California, Utah, and New York that participate in the National Patient-Centered Clinical Research Network (PCORnet). Patients were eligible to join if they were 18 years or older and had at least one clinical encounter after January 1, 2019. Recruitment lasted from November 2020 to February 2022.

### Participant-Reported Data

Upon enrolling, participants were asked to respond to baseline surveys on demographics, smoking history, and medical conditions. Participants were then administered follow-up surveys about exposure to, diagnosis of, and vaccination against COVID-19, among other questions seeking to understand both individual experience and population-level trends related to the pandemic. These surveys were housed in the Eureka research platform (University of California San Francisco, with funding from the National Institutes of Health) [16], which had web browser and smartphone functionality.

### EHR Data

For consenting and authorizing participants, EHR-limited datasets in the PCORnet Common Data Model format were extracted from the site-specific DataMarts maintained by all participating health systems [17]. The CCS study data extraction query was developed by Duke University programmers using SAS 9.4 software and distributed to all sites to run in their local environments against their DataMart. The query extracted clinical data with a 5-year lookback from the recruitment start date through the most recently available data. Sensitive diagnoses were filtered out, and only a minimum necessary subset of laboratory and medication records was extracted. Only patients for whom identities were algorithmically matched or manually verified were included in the final analytic cohort.

### Concordance Definitions

Among 34,908 participants where linkage was possible, we evaluated concordance in the following domains: demographics, baseline medical conditions, current smoker status, COVID-19 diagnosis, and COVID-19 vaccination. We chose variables that were conceptually similar between the participant-reported and EHR sources (MultimediaAppendices 1  2). Sex in both sources was defined as sex assigned at birth. Although gender identity was available from survey data, it was not available in EHR data and thus was not an eligible variable for concordance analysis. Race and ethnicity data abstracted from EHR data were populated according to health system practices. Race and ethnicity data abstracted from survey data were reported directly by study participants.

To promote comparability, measurement periods were aligned between data sources. Participants who had missing data in one or both sources were not considered for concordance analyses. Age data were not analyzed for concordance because a birthdate match between sources was a requirement for data to be considered for EHR data extraction; thus, discordant scenarios were inherently filtered out before analysis for this substudy.

For demographic, smoker status, and COVID-19 characteristics, the participant report was considered the criterion standard. For medical conditions, the EHR was considered the criterion standard.

### Statistical Approach

To test for marginal homogeneity between data sources, McNemar tests for paired nominal data were run on all 23 attributes, structured as dichotomous 2×2 contingency tables. Chi-square statistics and *P* values were calculated. A Bonferroni correction was applied to account for multiple comparisons, adjusting the significance threshold to .002 (.05/23). *P* values less than .001 were reported as *P*<.001.

For all domains, the following statistics were generated along with their 95% CI values: overall agreement (or overall accuracy), sensitivity, specificity, positive predictive value (PPV), negative predictive value (NPV), and Cohen κ. We used the following ranges for Cohen κ to describe observed agreement: strong (0.81‐1.00), good (0.61‐0.80), moderate (0.41‐0.60), fair (0.21‐0.40), poor (0.01‐0.20), and no agreement (<0) [18,19]. However, these ranges are to provide a guide, and the adequacy of the specific level of agreement should be considered specifically to the domain under consideration and the application to which it will be used.

Data were analyzed from December 2022 to July 2023 using SAS 9.4 software.

### Ethical Considerations

The CCS study, the protocol for which covered data analysis activities conducted for this substudy, was approved by the Western Institutional Review Board on November 5, 2020. Participants were informed of their right to withdraw at any time without any consequences. Digital informed consent was obtained from participants before surveys were collected. To maintain confidentiality, participants were not asked to provide their name, only their pre-assigned unique code to enable linkage to EHRs. Participants were not compensated for their time spent completing the surveys.

## Results

**Figure 1.**
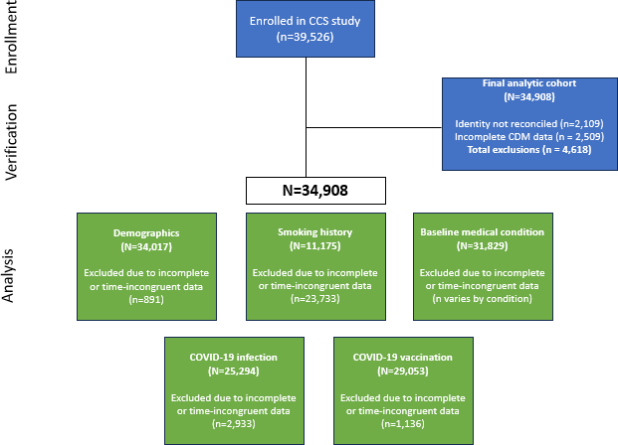
Enrollment diagram and final analytic cohort for the COVID-19 Citizen Science Study concordance substudy. CCS: COVID-19 Citizen Science Study; CDM: common data model.

A total of 39,526 patients enrolled in the study across 7 sites. After the exclusion of participants whose identity could not be verified and participants with partially or completely missing EHR data, 34,908 participants were included in the final analytic cohort (Figure 1). Descriptive statistics of the 34,017 participants who responded to the baseline demographic survey and results from the McNemar test for marginal homogeneity between data sources are summarized in Table 1. The median age of the sample was 57 (IQR 42‐68, range 18‐100) years according to the EHR. The sample was predominantly female and White according to both sources. The sample was classified as 6.6% (2,249/34,017) Hispanic in the EHR and 9.2% (3,124/34,017) Hispanic according to participant-reported data (*P*<.001).

**Table 1.** Comparison of demographic information derived from electronic health records and participant self-reports.

Variable	EHR^a^ (N=34,017)	Participant report (N=34,017)	*P* value^b^
Age (years)^c^			
Mean (SD)	54.7 (16.1)	—^d^	—
Median (IQR), range	57 (42‐68), 18-100	—	—
Sex, n (%)			
Female	21,217 (62.4)	21,176 (62.3)	.57
Male	12,780 (37.6)	12,742 (37.5)	
Refused or missing	20 (<1)	99 (<1)	
Race, n (%)			
American Indian or Alaska Native	104 (<1)	91 (<1)	.21
Asian, Native Hawaiian, or Pacific Islander	1797 (5.3)	2042 (6.0)	<.001
Black or African American	1286 (3.8)	1344 (4.0)	.001
White	27,054 (79.5)	27,744 (81.6)	<.001
Multiple races^e^	93 (<1)	1269 (3.7)	<.001
Other^f^	1039 (3.1)	1077 (3.2)	.33
Refused or missing	2644 (7.8)	450 (1.3)	<.001
Ethnicity, n (%)			
Hispanic	2249 (6.6)	3124 (9.2)	<.001
Non-Hispanic	28,903 (85.0)	30,528 (89.7)	
Refused or missing	2865 (8.4)	365 (1.1)	

a EHR: electronic health record.

b *P* values calculated by McNemar test.

c Age data were not analyzed for concordance because a date of birth match between sources was a requirement for data to be considered for EHR data extraction; thus, discordant scenarios were inherently filtered out before analysis for this substudy

d Not available.

e The category “Multiple Races” is a mapped value in the PCORnet Common Data Model, and no further detail was available. In participant-reported data, “Multiple Races” was defined as participants who responded to 2 or more non-missing race categories.

f In both data sources, there were no additional details for the “Other” categorization.

Statistically significant differences between the 2 data sources were detected for all characteristics except for the female category (*P*=.57), the American Indian or Alaska Native race category (*P*=.21), the “other” race category (*P*=.33), and diabetes (*P*=.17). The starkest absolute difference between data sources was for COVID-19 vaccination, in which 97.4% (28,291/29,053) of participants self-reported a vaccine while only 48.4% (14,076/29,053) had this documented in the EHR (Table 2).

**Table 2.** Comparison of medical conditions and COVID-19 history as reported in electronic health records and participant self-reports.

Variables	Participants, n	EHR^a^, n (%)	Participant report, n (%)	*P* value^b^
Smoking status	11,175	947 (8.5)	1396 (12.5)	<.001
Medical conditions				
Diabetes	31,744	3033 (9.6)	2979 (9.4)	.17
Hypertension	31,636	9440 (29.8)	10,953 (34.6)	<.001
Coronary artery disease or angina	31,573	2440 (7.7)	1941 (6.1)	<.001
Myocardial infarction	31,721	288 (0.9)	792 (2.5)	<.001
Congestive heart failure	31,686	689 (2.2)	545 (1.7)	<.001
Transient ischemic attack	31,659	533 (1.7)	945 (3)	<.001
Atrial fibrillation or flutter	31,495	1363 (4.3)	1769 (5.6)	<.001
Sleep apnea	31,075	2838 (9.1)	4902 (15.8)	<.001
COPD^c^	31,626	1469 (4.6)	4902 (3.5)	<.001
Asthma	31,738	3481 (11)	3150 (9.9)	<.001
Immunodeficiency	31,378	730 (2.3)	1611 (5.1)	<.001
Anemia	31,622	3838 (12.1)	3427 (10.8)	<.001
COVID-19				
Infection	25,294	2319 (9.2)	4446 (17.6)	<.001
Vaccination	29,053	14,076 (48.4)	28,291 (97.4)	<.001

a EHR: electronic health record.

b *P* values calculated by McNemar test.

c COPD: chronic obstructive pulmonary disease.

Agreement between EHR and participant-reported characteristics according to 5 proportionate measures (overall agreement, sensitivity, specificity, PPV, and NPV) and one statistic of interrater reliability (Cohen κ) are shown in Table 3 and Multimedia Appendix 3. Overall agreement was above 95% for all demographic characteristics, where the participant report was considered the criterion standard. Chance-corrected agreement (κ) was strong for most demographic characteristics except the “other” race category (κ=0.31) and current smoker status (κ=0.54). Sensitivity was 74% for the Hispanic characteristic, which translates to a relatively higher number of false negatives compared to other racial groups, 49.4% for current smoker status, and 26.6% for the “other” race category.

**Table 3.** Agreement between electronic health record– and participant-reported characteristics: overall agreement, κ statistic, and accuracy metrics.

Variable	Overall agreement, % (95% CI)	κ Statistic, % (95% CI)	Sensitivity, % (95% CI)	Specificity, % (95% CI)	PPV^a^, % (95% CI)	NPV^b^, % (95% CI)
Female	99.6 (99.5‐99.7)	0.99 (0.99‐0.99)	99.6 (99.5‐99.7)	99.4 (99.3‐99.6)	99.7 (99.6‐99.7)	99.4 (99.2‐99.5)
Non-Hispanic						
Asian American and Pacific Islander	99.2 (99.1‐99.3)	0.93 (0.92‐0.94)	92.0 (90.7‐93.3)	99.6 (99.6‐99.7)	93.9 (92.8‐95.1)	99.5 (99.4‐99.6)
Black	99.6 (99.5‐99.7)	0.94 (0.93‐0.95)	97.2 (96.2‐98.2)	99.7 (99.6‐99.7)	91.6 (90.0‐93.2)	99.9 (99.9‐99.9)
White	96.1 (95.9‐99.3)	0.87 (0.86‐0.88)	99.2 (99.0‐99.3)	83.3 (82.3‐84.3)	96.2 (95.9‐96.4)	95.9 (95.3‐96.4)
Other	96.9 (96.7‐97.1)	0.31 (0.27‐0.36)	26.6 (23.6‐29.6)	99.0 (98.8‐99.1)	42.7 (38.4‐47.0)	97.9 (97.7‐98.1)
Hispanic	97.9 (97.7‐98.0)	0.82 (0.81‐0.83)	74.0 (72.1‐75.9)	99.7 (99.6‐99.8)	94.9 (93.8‐96.0)	98.1 (97.9‐98.2)
Current smoker	91.4 (90.9‐91.9)	0.54 (0.52‐0.57)	49.4 (46.8‐52.1)	97.4 (97.1‐97.7)	72.9 (70.0‐75.7)	93.1 (92.6‐93.6)
Diabetes	95.1 (94.9‐95.3)	0.71 (0.70‐0.73)	73.5 (71.9‐75.1)	97.4 (97.2‐97.6)	74.8 (73.3‐76.4)	97.2 (97.0‐97.4)
Hypertension	85.0 (84.6‐85.4)	0.66 (0.64‐0.68)	82.9 (82.1‐83.6)	85.9 (85.4‐86.4)	71.4 (70.6‐72.3)	92.2 (91.8‐92.6)
Coronary artery disease/angina	94.0 (93.7‐94.3)	0.54 (0.52‐0.56)	51.0 (49.0‐53.0)	97.6 (97.4‐97.8)	64.1 (62.0‐66.2)	96.0 (95.7‐96.2)
Myocardial infarction	97.6 (97.5‐97.8)	0.30 (0.25‐0.35)	57.6 (51.9‐63.3)	98.0 (97.9‐98.2)	21.0 (18.1‐23.8)	99.6 (99.5‐99.7)
Congestive heart failure	98.0 (97.9‐98.2)	0.48 (0.44‐0.52)	44.1 (40.4‐47.8)	99.2 (99.1‐99.3)	55.8 (51.6‐60.0)	98.8 (98.6‐98.9)
Transient ischemic attack	97.6 (97.4‐97.7)	0.47 (0.43‐0.50)	66.2 (62.2‐70.2)	98.1 (97.9‐98.3)	37.4 (34.3‐40.4)	99.4 (99.3‐99.5)
Atrial fibrillation	97.0 (96.8‐97.2)	0.68 (0.66‐0.70)	80.3 (78.2‐82.4)	97.8 (97.6‐98.0)	61.8 (59.6‐64.1)	99.1 (99.0‐99.2)
Sleep apnea	90.4 (90.0‐90.7)	0.56 (0.55‐0.58)	83.5 (82.2‐84.9)	91.0 (90.7‐91.4)	48.4 (47.0‐49.8)	98.2 (98.1‐98.4)
COPD^c^	95.2 (95.0‐95.5)	0.39 (0.36‐0.42)	36.7 (34.2‐39.2)	98.1 (97.9‐98.3)	48.4 (45.5‐51.4)	97.0 (96.8‐97.1)
Asthma	90.2 (89.9‐90.6)	0.48 (0.46‐0.50)	50.8 (49.1‐52.5)	95.1 (94.9‐95.4)	56.1 (54.4‐57.9)	94.0 (93.7‐94.3)
Immunodeficiency	94.6 (94.4‐94.9)	0.26 (0.22‐0.29)	45.1 (41.5‐48.7)	95.8 (95.6‐96.0)	20.4 (18.5‐22.4)	98.7 (98.5‐98.8)
Anemia	85.0 (84.6‐85.4)	0.26 (0.24‐0.28)	32.8 (31.3‐34.3)	92.2 (91.9‐92.5)	36.7 (35.1‐38.3)	90.8 (90.5‐91.2)
COVID-19 infection	84.6 (84.1‐85.0)	0.34 (0.33‐0.36)	32.2 (30.9‐33.6)	95.8 (95.5‐96.0)	61.8 (59.8‐63.8)	86.9 (86.5‐87.3)
COVID-19 vaccination	51.0 (50.4‐51.6)	0.05 (0.04‐0.06)	49.7 (49.1‐50.3)	98.2 (97.2‐99.1)	99.9 (99.9‐99.9)	5.0 (4.6‐5.3)

a PPV: positive predictive value.

b NPV: negative predictive value.

c COPD: chronic obstructive pulmonary disease.

The criterion standard for baseline medical conditions was the EHR. Overall agreement, specificity, and NPV between data sources was above 85% for all baseline medical conditions, although there was heterogeneity in sensitivity, PPV, and chance-corrected agreement (κ). Sensitivity ranged from 32.8 (95% CI 31.3‐34.3) to 83.5% (95% CI 82.2‐84.9), being lowest for anemia and highest for sleep apnea. PPV ranged from 20.4% (95% CI 18.4‐22.4) to 74.8% (95% CI 73.3‐76.4), being lowest for immunodeficiency and highest for diabetes. Finally, chance-corrected agreement (κ) was good for 3 of 12 compared baseline medical conditions, moderate for 5, and fair for 4. Chance-corrected agreement (κ) ranged from 0.26 (95% CI 0.22‐0.29) to 0.71 (95% CI 0.70‐0.73) for non-HIV immunodeficiency and diabetes, respectively.

The criterion standard for COVID-19 variables was participant-reported data. While chance-corrected agreement was fair for COVID-19 infection (κ=0.34), it was poor for COVID-19 vaccination (κ=0.05). Of 25,294 participants whose COVID-19 infection data could be compared, there were 3899 cases of discordance, 77.3% (3013/3899) of which were classified as the participant reporting a COVID-19 diagnosis but this not being reflected in the EHR. Similarly, of the 29,053 participants whose COVID-19 vaccine data could be compared, there were 14,243 cases of discordance, 99.9% (14,229/14,243) of which were classified as the participant reporting a COVID-19 vaccine but this not being reflected in the EHR.

## Discussion

### Principal Results

We evaluated the concordance survey data from participants enrolled in a COVID-19 study and their linked EHR data. We had four main findings: (1) sensitivity and chance-corrected agreement were strong for all demographic characteristics except for Hispanic ethnicity, the “other” race category, and current smoker status, indicating that a relatively lower proportion of patients were correctly identified in the EHR as such in comparison to other traits; (2) when the EHR was the criterion standard, as we considered it to be for medical conditions, specificity and NPV were higher than corresponding sensitivity and PPV, suggesting that patients were better able to report in concordance with their EHR the absence of a medical condition rather than the presence of one; (3) chance-corrected agreement, sensitivity, and PPV varied widely for medical conditions, with no clear pattern emerging as to which types were more likely to be self-reported in concordance with the EHR; and (4) COVID-19 infection and vaccination had relatively low chance-corrected agreement and overall agreement compared to most demographic traits and many medical conditions, along with very low sensitivity, indicating that these health events are poorly captured in patients’ EHRs.

Our findings suggest the need for improvements to point-of-care capture of patient demographic traits and COVID-19 infection and vaccination history, patient education about their medical conditions, and linkage to external data sources in EHR-only pragmatic research. Our results indicating specifically that the capture of Hispanic ethnicity and “other” race category is not as sensitive as other race categories demonstrates that current point-of-care processes for collecting racial and ethnic information from patients may be insufficient, regardless of whether such data points are captured by a clinician, administrative worker, or the patients themselves. It is critical to have granular and accurate capture of patient race and ethnicity to provide the most culturally sensitive clinical care. Similarly, our findings that captured COVID-19 health events were relatively discordant between data sources, suggesting an interruption of health data flow back to the EHR. This could originate from improper integration of COVID-19 testing and vaccination data sources, especially when these events happen outside of the health system, resulting in health care providers not having the most up-to-date information about the health status of their patients. Finally, the lower specificity and NPV of medical conditions when compared to their corresponding sensitivity and PPV suggest that (1) EHRs may not be capturing medical conditions, especially those that are pre-existing; and (2) patients may not be aware that they have certain medical conditions, most marked for conditions like anemia and COPD, both of which showed only about a third of patients reporting the presence of these conditions in concordance with their EHRs. Comprehensive and customized patient education and communication are suggested to support self-management of medical conditions and patient autonomy outside the point of care. Further, and for the sake of research integrity, those engaging in EHR-only research should make attempts to access and query as many views and tables as is feasible to properly categorize a patient as having or not having a medical condition.

Increasingly, novel research designs rely on the integration of multiple data sources to answer research questions. The COVID-19 pandemic accelerated already growing interest in real-world data use cases [20], including leveraging existing EHR data and bringing research directly to people through participant-facing portals. Direct-to-participant research has numerous benefits, including potential for greater geographic reach and diversity, lower participant burden with few or no in-person visits, and platforms that enable capture of relevant patient-centered endpoints [21]. These strengths complement those of EHR data, which, through national networks like PCORnet, can be standardized into research-grade data to facilitate rapid insights into key clinical outcomes [22]. To our knowledge, this is the first study to examine patterns of concordance across participant-reported and EHR data in the context of COVID-19.

Our finding that participants self-reported COVID-19 infection and vaccination at higher rates than what was evident in their clinical records illustrates the fragmented nature of real-world data. Ongoing work to enhance the quality and reliability of EHR data in the context of COVID-19, including network-level curation [22], linkage to external sources where appropriate (eg, state vaccine [23] or policy [24] databases), and systematic phenotype development and testing [25], is critical to maximizing the research value of these data. In parallel, the implementation of best practices to enhance the validity of participant reports, including stakeholder engagement in survey design, readability assessments, and cultural and linguistic adaptation, is essential to enhancing the reliability of findings from participant-facing research [26,27]. Our findings are broadly consistent with those from prior studies, suggesting that fitness-for-use depends on context [28,29]. We found that no single data source may be appropriate for EHR-based pragmatic research, consistent with prior work illustrating the potential biases that can arise in participant-reported data and how they vary [30-33].

### Limitations

Several limitations to our study are worth noting. First, the CCS study comprises participants who were mostly White and female. Therefore, results may not generalize to broader populations. Second, diversity within minority communities can make both responding to survey questions and the identification of race challenging for patients and clinicians, respectively, a fact that may skew findings from our demographic analyses. Third, participant-reported COVID-19 variables were not validated and are subject to reporting and recall bias. Fourth, we observed some attrition in reporting over time, which could lead to selection bias in analyses of longitudinal outcomes. Finally, we used EHR data for this concordance analysis, which may not represent all medical encounters for a given participant and which may not be of the highest or most accurate quality. EHR data used in this analysis were not linked to claims data from pharmacies, which are a major administrator of COVID-19 vaccines. Particularly for outcomes that are generally observed outside of the hospital, linkage to external data sources is likely warranted.

### Conclusions

We found that the integration of multiple data sources to investigate COVID-19 research questions enhances the capture of key elements but also introduces opportunities for disagreement. Future studies that leverage linked data should evaluate the concordance of overlapping elements and report levels of agreement. Transparent reporting will contribute to a broader understanding of data reliability and relevance and support future strategies to improve fitness-for-use of real-world data.

## Supplementary material

10.2196/58097 Multimedia Appendix 1 Description of concordance definitions by electronic health record and participant report.

10.2196/58097 Multimedia Appendix 2 Clinical codes used in medical condition and COVID-19 phenotypes.

10.2196/58097 Multimedia Appendix 3 2x2 contingency tables for all concordance domains.

## Abbreviations

CCS: COVID-19 Citizen Science Study
EHR: electronic health record
NPV: negative predictive value
PCORnet: National Patient-Centered Clinical Research Network
PPV: positive predictive value
