# Supplementary material for: Concordance Between Survey and Electronic Health Record Data in the COVID-19 Citizen Science Study: Retrospective Cohort Analysis
Source: JMIR Form Res. 2025 Jul 28;9:e58097. doi: 10.2196/58097 (PMC12303549; doi:10.2196/58097)
Supplement: Multimedia Appendix 1 [file formative-v9-e58097-s001.docx]

**Table S1.**

| Variable | Electronic heath record | Participant report |
| --- | --- | --- |
| Sex, race, and ethnicity | Participant had indication of demographic characteristic, captured statically | Participant responded affirmatively to question asking about demographic characteristic |
| Current smoker | Participant had indication of being a current (everyday, some days) light or heavy tobacco or cigarette smoker at the most recent, non-missing vital capture | Participant responded affirmatively to question asking if they had smoked any tobacco product in the last month |
| COVID-19 infection | Participant had at least one ICD-10 diagnosis code indicating COVID-19 infection in all available data,  **OR**  Participant had at least one SARS-CoV-2 laboratory resulted positive or detected prior to the affirmative survey response date | Participant responded affirmatively to any question at any point during survey collection about having previously received a COVID-19 diagnosis or positive laboratory test indicating presence of SARS-CoV-2 virus |
| COVID-19 vaccination | Participant had at least one CPT/HCPCS or CVX code indicating COVID-19 vaccination prior to most recent affirmative survey response | Participant responded affirmatively to any question at any point during survey collection about having previously received a COVID-19 primary series or booster vaccination |
| Medical conditions | Participant had at least one ICD-10 diagnosis code for medical condition in all available data | Participant responded affirmatively to baseline survey question about having ever been diagnosed with or treated by a medical professional for the medical condition |
