# Supplementary material for: Concordance Between Survey and Electronic Health Record Data in the COVID-19 Citizen Science Study: Retrospective Cohort Analysis
Source: JMIR Form Res. 2025 Jul 28;9:e58097. doi: 10.2196/58097 (PMC12303549; doi:10.2196/58097)
Supplement: Multimedia Appendix 2 [file formative-v9-e58097-s002.docx]

**Table S1.**

| **Characteristics** | **Ontology** | **Codes** |
| --- | --- | --- |
| Diabetes | ICD-10 | E08x, E09x, E10x, E11x, E13x |
| Hypertension | ICD-10 | I10x, I11x, I12x, I13x, I15x, I16x |
| Coronary artery disease/angina | ICD-10 | I2510, I25110, I25111, I25112, I25118, I25119, I252, I253, I2541, I2542, I255, I256, I25700, I25701, I25702, I25708, I25709, I25710, I25711, I25712, I25718, I25719, I25720, I25721, I25722, I25728, I25729, I25730, I25731, I25732, I25738, I25739, I25750, I25751, I25752, I25758, I25759, I25760, I25761, I25762, I25768, I25769, I25790, I25791, I25792, I25798, I25799, I25810, I25811, I25812, I2582, I2583, I2584, I2589, I259, I200, I201, I202, I208, I209 |
| Myocardial infarction | ICD-10 | I2101, I2102, I2109, I2111, I2119, I2121, I2129, I213, I214, I219, I21A1, I21A9 |
| Congestive heart failure | ICD-10 | I501, I5020, I5021, I5022, I5023, I5030, I5031, I5032, I5033, I5040, I5041, I5042, I5043, I50810, I50811, 50812, I50813, I50814, I5082, I5083, I5084, I5089, I509 |
| Transient ischemic attack | ICD-10 | I6300, I63011, I63012, I63013, I63019, I6302, I63031, I63032, I63033, I63039, I6309, I6310, I63111, I63112, I63113, I63119, I6312, I63131, I63132, I63133, I63139, I6319, I6320, I63211, I63212, I63213, I63219, I6322, I63231, I63232, I63233, I63239, I6329, I6330, I63311, I63312, I63313, I63319, I63321, I63322, I63323, I63329, I63331, I63332, I63333, I63339, I63341, I63342, I63343, I63349, I6339, I6340, I63411, I63412, I63413, I63419, I63421, I63422, I63423, I63429, I63431, I63432, I63433, I63439, I63441, I63442, I63443, I63449, I6349, I6350, I63511, I63512, I63513, I63519, I63521, I63522, I63523, I63529, I63531, I63532, I63533, I63539, I63541, I63542, I63543, I63549, I6359, I636, I6381,I6389,I639, G450, G451,G452,G453, G454, G458,G459 |
| Atrial fibrillation | ICD-10 | I480, I4811, I4819, I4820, I4821, I483, I484, I4891, I4892 |
| Sleep apnea | ICD-10 | G4733, G4734, G4735, G4736, G4737, G4739 |
| COPD | ICD-10 | J40, J410, J411, J418, J42, J430, J431, J432, J438, J439, J440, J441, J449 |
| Asthma | ICD-10 | J4520, J4521, J4522, J4530, J4531, J4532, J4540, J4541, J4542, J4550, J4551, J4552, J45901, J45902, J45909, J45990, J45991, J45998 |
| Immunodeficiency (excluding HIV) | ICD-10 | D800, D801, D802, D803, D804, D805, D806, D807, D808, D809, D810, D811, D812, D8130, D8131, D8132, D8139, D814, D815, D816, D817, D81810, D81818, D81819, D8182, D8189, D819, D820, D821, D822, D823, D824, D828, D829, D830, D831, D832, D838, D839, D840, D841, D8481, D8482, D84822, D8489, D849, D890, D891, D892, D893, D8940, D8941, D8942, D8943, D8944, D8949, D89810, D89811, D89812, D89813, D8982, D89831, D89832, D89833, D89834, D89835, D89839, D8989, D899 |
| Anemia | ICD-10 | D500, D501, D508, D509, D510, D511, D512, D513, D518, D519, D520, D521, D528, D529, D530, D531, D532, D538, D539, D550, D551, D5521, D5529, D553, D558, D559, D560, D561, D562, D563, D564, D565, D568, D569, D5700, D5701, D5702, D5703, D5709, D571, D5720,D57211, D57212, D57213, D57218, D57219, D573, D5740, D57411, D57412, D57413, D57418, D57419, D5742, D57431, D57432, D57433, D57438, D57439, D5744, D57451, D57452, D57453, D57458, D57459, D5780, D57811, D57812, D57813, D57818, D57819, D580, D581, D582, D588, D589, D590, D5910, D5911, D5912, D5913, D5919, D592, D5930, D5931, D5932, D5939, D594, D595, D596, D598, D599, D600, D601, D608, D609, D6101, D6109, D611, D612, D613, D61810, D61811, D61818, D6182, D6189, D619, D62, D630, D631, D638, D640, D641, D642, D643, D644, D6481, D6489, D649 |
| Covid-19 infection | ICD-10 | B34.2, B97.2, B97.21, B97.29, J12.81, U04.9, U07.1, U07.2, U09.9, Z86.16 |
|  | LOINC | 94503-0, 94504-8, 96118-5, 94507-1 ,94508-9, 94547-7, 94562-6, 94563-4, 94564-2, 94661-6, 94761-4, 94762-2, 94768-9, 95125-1, 95411-5, 95416-4, 95542-7, 95825-6, 94505-5, 94506-3, 94720-0, 94769-7, 95410-7, 95427-1, 95428-9, 95429-7, 96742-2, 94558-4, 95209-3, 96119-3, 97097-0, 94763-0, 94764-8, 94306-8, 94531-1, 95380-2, 95422-2, 95941-1, 95942-9, 96094-8, 96894-1, 97099-6, 94307-6, 94308-4, 94309-2, 94310-0, 94314-2, 94315-9, 94316-7, 94500-6, 94502-2, 94532-9, 94533-7, 94534-5, 94559-2, 94565-9, 94639-2, 94640-0, 94641-8, 94647-5, 94660-8, 94756-4, 94757-2, 94758-0, 94759-8, 94760-6, 94765-5, 94766-3, 94767-1, 94822-4, 94845-5, 95406-5, 95409-9, 95423-0, 95424-8, 95425-5, 95608-6, 95609-4, 95823-1, 95824-9, 96091-4, 96120-1, 96121-9, 96122-7, 96123-5, 96448-6, 96741-4, 96763-8, 96765-3, 96957-6, 96958-4, 96986-5, 97098-8, 94311-8, 94312-6, 94313-4, 94509-7, 94510-5, 94511-3, 94642-6, 94643-4, 94644-2, 94645-9, 94646-7, 94745-7, 94746-5, 94819-0, 95521-1, 95522-9, 96764-6, 97104-4 |
| Covid-19 vaccination | CVX | 207, 208, 211, 212, 217, 218, 219, 221, 227, 228, 229, 230, 300, 301, 302 |
|  | NDC | 00069100002, 00069100003, 00069202501, 00069202510, 00069202525, 00310122210, 59267007801, 59267007804, 59267030401, 59267056501, 59267060901, 59267100001, 59267100002, 59267100003, 59267102501, 59267102502, 59267102503, 59267102504, 59267105501, 59267105502, 59267105504, 59267140401, 59676058005, 59676058015, 80631010001, 80631010010, 80777010011, 80777010099, 80777027310, 80777027315, 80777027398, 80777027399, 80777027505, 80777027599, 80777027705, 80777027799, 80777027905, 80777027999, 80777028005, 80777028099, 80777028205, 80777028302 |
|  | CPT/HCPCS | 91302, 91300, 91301, 91302, 91303, 91304, 91305, 91306, 91307 91308, 91309, 91311, 91312, 91313, 91314, 91315, 91316, 91317, 0001A, 0002A, 0003A, 0004A, 0011A, 0012A, 0013A, 0021A, 0022A, 0031A, 0034A, 0041A, 0042A, 0044A, 0051A, 0052A, 0053A, 0054A, 0064A, 0071A, 0072A, 0073A, 0074A, 0081A, 0082A, 0083A, 0091A, 0092A, 0093A, 0094A, 0104A, 0111A, 0112A, 0113A, 0124A, 0134A, 0144A, 0154A, 0164A, 0173A, 0174A, 91310, D1701, D1702, D1703, D1704, D1707, D1708, D1709, D1710, D1711, D1712, D1713, D1714, M0201 |
