# Supplementary material for: Concordance Between Survey and Electronic Health Record Data in the COVID-19 Citizen Science Study: Retrospective Cohort Analysis
Source: JMIR Form Res. 2025 Jul 28;9:e58097. doi: 10.2196/58097 (PMC12303549; doi:10.2196/58097)
Supplement: Multimedia Appendix 3 [file formative-v9-e58097-s003.docx]

**Table S1.**

| Female | | | |  | Diabetes | | | |
| --- | --- | --- | --- | --- | --- | --- | --- | --- |
|  | EHR/CDM |  |  |  |  | EHR/CDM |  |  |
| Patient Report | Yes | No | Total |  | Patient Report | Yes | No | Total |
| Yes | 21087 | 80 | 21167 |  | Yes | 2229 | 750 | 2979 |
| No | 73 | 12660 | 12733 |  | No | 804 | 27961 | 28765 |
| Total | 21160 | 12740 | 33900 |  | Total | 3033 | 28711 | 31744 |
|  |  |  |  |  |  |  |  |  |
| Male | | | |  | Hypertension | |  |  |
|  | EHR/CDM |  |  |  |  | EHR/CDM |  |  |
| Patient Report | Yes | No | Total |  | Patient Report | Yes | No | Total |
| Yes | 12660 | 73 | 12733 |  | Yes | 7823 | 3130 | 10953 |
| No | 80 | 21087 | 21167 |  | No | 1617 | 19066 | 20683 |
| Total | 12740 | 21160 | 33900 |  | Total | 9440 | 22196 | 31636 |
|  |  |  |  |  |  |  |  |  |
| Nonhispanic AAPI | | | |  | Coronary blockage/angina | | |  |
|  | EHR/CDM |  |  |  |  | EHR/CDM |  |  |
| Patient Report | Yes | No | Total |  | Patient Report | Yes | No | Total |
| Yes | 1549 | 134 | 1683 |  | Yes | 1244 | 697 | 1941 |
| No | 100 | 27520 | 27620 |  | No | 1196 | 28436 | 29632 |
| Total | 1649 | 27654 | 29303 |  | Total | 2440 | 29133 | 31573 |
|  |  |  |  |  |  |  |  |  |
| Nonhispanic Black | | | |  | Myocardial infarction | |  |  |
|  | EHR/CDM |  |  |  |  | EHR/CDM |  |  |
| Patient Report | Yes | No | Total |  | Patient Report | Yes | No | Total |
| Yes | 1013 | 29 | 1042 |  | Yes | 166 | 626 | 792 |
| No | 93 | 28168 | 28261 |  | No | 122 | 30807 | 30929 |
| Total | 1106 | 28197 | 29303 |  | Total | 288 | 31433 | 31721 |
|  |  |  |  |  |  |  |  |  |
| Nonhispanic White | | | |  | Congestive heart failure | | |  |
|  | EHR/CDM |  |  |  |  | EHR/CDM |  |  |
| Patient Report | Yes | No | Total |  | Patient Report | Yes | No | Total |
| Yes | 23480 | 200 | 23680 |  | Yes | 304 | 241 | 545 |
| No | 940 | 4683 | 5623 |  | No | 385 | 30756 | 31141 |
| Total | 24420 | 4883 | 29303 |  | Total | 689 | 30997 | 31686 |
|  |  |  |  |  |  |  |  |  |
|  |  |  |  |  |  |  |  |  |
| Nonhispanic Other | | | |  | Stroke |  |  |  |
|  | EHR/CDM |  |  |  |  | EHR/CDM |  |  |
| Patient Report | Yes | No | Total |  | Patient Report | Yes | No | Total |
| Yes | 220 | 608 | 828 |  | Yes | 353 | 592 | 945 |
| No | 295 | 28180 | 28475 |  | No | 180 | 30534 | 30714 |
| Total | 515 | 28788 | 29303 |  | Total | 533 | 31126 | 31659 |
|  |  |  |  |  |  |  |  |  |
|  |  |  |  |  |  |  |  |  |
| Hispanic | | | |  | Atrial flutter/fibrillation | | |  |
|  | EHR/CDM |  |  |  |  | EHR/CDM |  |  |
| Patient Report | Yes | No | Total |  | Patient Report | Yes | No | Total |
| Yes | 1531 | 538 | 2069 |  | Yes | 1094 | 675 | 1769 |
| No | 82 | 27152 | 27234 |  | No | 269 | 29457 | 29726 |
| Total | 1613 | 27690 | 29303 |  | Total | 1363 | 30132 | 31495 |
|  |  |  |  |  |  |  |  |  |
|  |  |  |  |  |  |  |  |  |
| Current smoker | |  |  |  | Apnea |  |  |  |
|  | EHR/CDM |  |  |  |  | EHR/CDM |  |  |
| Patient Report | Yes | No | Total |  | Patient Report | Yes | No | Total |
| Yes | 690 | 706 | 1396 |  | Yes | 2371 | 2531 | 4902 |
| No | 257 | 9522 | 9779 |  | No | 467 | 25706 | 26173 |
| Total | 947 | 10228 | 11175 |  | Total | 2838 | 28237 | 31075 |
|  |  |  |  |  |  |  |  |  |
|  |  |  |  |  |  |  |  |  |
| Covid-19 indication | |  |  |  | Chronic obstructive pulmonary disease | | | |
|  | EHR/CDM |  |  |  |  | EHR/CDM |  |  |
| Patient Report | Yes | No | Total |  | Patient Report | Yes | No | Total |
| Yes | 1433 | 3013 | 4446 |  | Yes | 539 | 574 | 1113 |
| No | 886 | 19962 | 20848 |  | No | 930 | 29583 | 30513 |
| Total | 2319 | 22975 | 25294 |  | Total | 1469 | 30157 | 31626 |
|  |  |  |  |  |  |  |  |  |
| Covid-19 vaccination | | | |  |  |  |  |  |
|  | EHR/CDM |  |  |  | Asthma |  |  |  |
| Patient Report | Yes | No | Total |  |  | EHR/CDM |  |  |
| Yes | 14062 | 14229 | 28291 |  | Patient Report | Yes | No | Total |
| No | 14 | 748 | 762 |  | Yes | 1768 | 1382 | 3150 |
| Total | 14076 | 14977 | 29053 |  | No | 1713 | 26875 | 28588 |
|  |  |  |  |  | Total | 3481 | 28257 | 31738 |
|  | | | |  |  |  |  |  |
|  |  | |  |  |  |  |  |  |
|  |  |  |  |  | Immunodeficiency (not HIV) | | |  |
|  |  |  |  |  |  | EHR/CDM |  |  |
|  |  |  |  |  | Patient Report | Yes | No | Total |
|  |  |  |  |  | Yes | 329 | 1282 | 1611 |
|  |  |  |  |  | No | 401 | 29366 | 29767 |
|  |  |  |  |  | Total | 730 | 30648 | 31378 |
|  |  |  |  |  |  |  |  |  |
|  |  |  |  |  |  |  |  |  |
|  |  |  |  |  | Anemia |  |  |  |
|  |  |  |  |  |  | EHR/CDM |  |  |
|  |  |  |  |  | Patient Report | Yes | No | Total |
|  |  |  |  |  | Yes | 1258 | 2169 | 3427 |
|  |  |  |  |  | No | 2580 | 25615 | 28195 |
|  |  |  |  |  | Total | 3838 | 27784 | 31622 |
